# NMR Insights into Folding and Self-Association of *Plasmodium falciparum* P2

**DOI:** 10.1371/journal.pone.0036279

**Published:** 2012-05-02

**Authors:** Pushpa Mishra, Sudipta Das, Lata Panicker, Madhusoodan V. Hosur, Shobhona Sharma, Ramakrishna V. Hosur

**Affiliations:** 1 Department of Chemical Sciences, Tata Institute of Fundamental Research, Mumbai, India; 2 Department of Biological Sciences, Tata Institute of Fundamental Research, Mumbai, India; 3 Department of Solid State Physics Division, Bhabha Atomic Research Centre, Mumbai, India; 4 UM-DAE Centre for Excellence in Basic Sciences, Mumbai University Campus, Mumbai, India; MRC National Institute for Medical Research, United Kingdom

## Abstract

The eukaryotic 60S-ribosomal stalk is composed of acidic ribosomal proteins (P1 and P2) and neutral protein P0, which are thought to be associated as a pentameric structure, [2P1, 2P2, P0]. *Plasmodium falciparum* P2 (PfP2) appears to play additional non-ribosomal functions associated with its tendency for homo-oligomerization. Recombinant bacterially expressed PfP2 protein also undergoes self-association, as shown by SDS-PAGE analysis and light scattering studies. Secondary structure prediction algorithms predict the native PfP2 protein to be largely helical and this is corroborated by circular dichroism investigation. The ^1^H-^15^N HSQC spectrum of native P2 showed only 43 cross peaks compared to the expected 138. The observed peaks were found to belong to the C-terminal region, suggesting that this segment is flexible and solvent exposed. In 9 M urea denaturing conditions the chain exhibited mostly non-native β structural propensity. ^15^N Relaxation data for the denatured state indicated substantial variation in ms-µs time scale motion along the chain. Average area buried upon folding (AABUF) calculations on the monomer enabled identification of hydrophobic patches along the sequence. Interestingly, the segments of slower motion in the denatured state coincided with these hydrophobic patches, suggesting that in the denatured state the monomeric chain undergoes transient hydrophobic collapse. The implications of these results for the folding mechanism and self-association of PfP2 are discussed.

## Introduction

The lateral flexible stalk of the large ribosomal subunit, a peculiar region made up of several proteins that is found in almost all ribosomes anchored to a conserved region of the 28S (23S) rRNA is termed the GTPase-associated domain or GTPase center [1]. The structure of the ribosomal GTPase within the large subunit is not resolved by X-ray crystallography, probably because of its high flexibility. Biochemical evidence shows that a pentameric acidic protein complex bound to the GTPase center forms a functional component in the ribosome [2]. The stalk composition varies across the biological kingdoms; in prokaryotes the stalk is comprised of proteins L10, L7/L12 forming either a pentameric L10-(L7/L12)_2_ or a heptameric L10-(L7/L12)_3_ complex [2], [3]. In higher eukaryotes the proteins P1 and P2 interact with P0 forming a pentameric P0-(P1/P2)_2_ complex, and are collectively known as the P-proteins [4], [5]. The stoichiometry and topography have been confirmed by many deletion and cross-linking experiments which predict that P1 and P2 form homodimers [6], whilst many other genetic, biochemical studies and also yeast two-hybrid experiments show that the P1–P2 heterodimer is linked to P0 to form a pentameric structure, P0-(P1–P2)_2_ [7]. The P proteins are known to contribute towards the structure of the stalk-domain of the large ribosomal subunit. It has been shown in prokaryotes that L7/L12 is involved in binding translation factors to the ribosome to stimulate GTP hydrolysis through stabilization of the GTPase conformation [8]. Through cryoelectron microscopy these complexes are observed as a lateral protuberance of the large ribosomal subunit and play an important role in translation elongation [9], [10]. This assembly is known for large changes of conformation during the different steps of the elongation cycle and has high intrinsic flexibility [11]. The precise details of the mechanism are still not understood. The structure of the isolated bacterial L7/L12 complex has been solved by X-ray crystallography and three domains were identified, which include a short alpha-helical N-terminal domain, an intermediary (hinge) domain, and a C-terminal domain found to bind elongation factors [12], [13].

Recently the crystal structure of the eukaryotic 60S ribosomal subunit from *Tetrahymena thermophila* in complex with elF6 has been reported [14]. However, in this structure there are no details about the stalk elements. Although the primary amino acid sequences of several eukaryotic P-proteins are known, structural details are relatively scarce. The recently elucidated solution structure of N-terminal region of the human P2 protein shows that the P2 structure is very distinct form that of the bacterial functional orthologue L12 [15]. Each of the three ribosomal P-proteins is separately conserved across several eukaryotic species and, in particular, the acidic C-terminal domain is conserved even among the P-proteins [16]. It had been reported that the N-terminal regions of eukaryotic acidic phosphoproteins P1 and P2 are crucial for heterodimerization and assembly into the ribosomal GTPase center [17]. The *Plasmodium* P2 protein also plays additional roles in parasite cell division (Das et al., communicated manuscript). Malaria parasites resident inside erythrocytes are confined to a parasitophorous vacuole. In addition to its presence in this parsitophorous vacuole, the Plasmodium P2 protein is also found on the infected-erythrocyte surface for 6–8 hrs during early schizogony, at the onset of cell-division. Treatment with a panel of anti-PfP2-specific monoclonal antibodies causes an arrest of such infected cells at the first nuclear division, indicating an unusual role of the P2 protein in parasite cell division. It points to complexity in the signaling cascade between the P2 protein at the infected-red cell surface and the nucleus of the parasite across several membrane layers. The P2 protein found at the red cell surface occurs exclusively as SDS-resistant homotetramers, as observed through mass spectrometric determinations of immunoprecipitated preparations of parasite infected red cell ghost membrane preparations.

Structural contributions of the P-proteins to the ribosome have best been addressed in the yeast *Saccharomyces cerevisiae* and the rat [18], [19], [20]. It has been shown earlier in yeast [21], [22] that isolated P2 forms a homodimer, but in presence of the P1 forms a more stable P1/P2 heterodimer [15]. Therefore P1/P2 heterodimers may play an important role in the assembly of the ribosomal stalk [23]. However, whether homo-oligomerization of either P1 or P2 is important in higher eukaryotes is not yet clear.

In *Plasmodium*, P2 homo-oligomerization is associated with its localization to the infected RBC surface, where it appears to play an important role in *Plasmodial* nuclear division (Das *et al.*, communicated manuscript). During the development in erythrocytes the P2 protein within the parasite body remains largely monomeric, as detected by SDS-PAGE, until the onset of cell division. At that point a large number of SDS-resistant higher oligomers of P2 protein are detected indicating that homo-oligomerization of P2 protein is developmentally regulated in *Plasmodium*. At the infected-erythrocyte surface, however, only the SDS-resistant homo-tetrameric form of P2 is found, pointing to a role of tetrameric P2 at the infected red cell surface. The occurrence of oligomers of P2 protein diminishes as the cell division proceeds to completion.

Thus the propensity to undergo self- or hetero-association may be important for the *Plasmodium* P-proteins. Self-association would be intimately connected with the folding and consequent surface properties of the polypeptide chain. Therefore, understanding the folding mechanism of these proteins is a relevant field of investigation.

Three different models, namely, the framework model [24], [25], [26], [27], hydrophobic collapse [28], [29], [30] and nucleation-condensation [31], [32], [33] have been developed to describe protein folding mechanisms. In the framework model, local regular secondary structure is formed early and then the segments assemble into the native tertiary structure by diffusional processes. In the hydrophobic collapse model non-polar residues combine to form a compact object prior to secondary structure formation. Finally, in the nucleation condensation model the protein chain forms a diffuse folding nucleus of a few adjacent residues which have some relevant secondary structure propensity, providing a platform for further folding.

In the case of P2, the high susceptibility to self-association hampers folding investigations. In this scenario a plausible strategy to understand folding is to denature the protein – which also dissociates the oligomers – and then characterize structural propensities and dynamics characteristics along the chain in the denatured state. Such characteristics have been known to throw light on folding initiation sites and structural transitions that occur when appropriate conditions of folding are provided [34], [35], [36].

*In vivo*, the newly synthesized polypeptide chain is in some kind of denatured state. The precise characteristics of the state vary according to the cell type and the intra-cellular environment. It is known that the properties of the denatured state and the solution conditions significantly influence the protein folding pathway and whether the end product is a properly folded or a misfolded state, which would have great impact on the function of the protein [37], [38], [39], [40]. In this view it is important to understand the characteristics of the denatured state.

While studying denatured states of proteins inside cells has remained a challenging task, useful insights have been derived on the biophysical aspects of folding, by studying denatured states created by chemical denaturants such as guanidine-HCl, sodium dodecyl sulfate, urea, organic solvents, etc. This allows sampling of the conformational space available to different polypeptide chains. Indeed all of these states are different [41], [42], [43]. It may be that some of these states would be populated under *in vivo* conditions, and hence step-wise studies of various unfolding perturbations can be taken to provide insight into the numerous trajectories that may be accessed by the polypeptide chain in the course of its folding to the native form.

With this concept in mind, we report here structural and dynamics characterization of the native oligomeric and the denatured monomeric forms of *Plasmodium falciparum* P2 (PfP2), which represent the two ends of the folding funnel. From various biophysical methods we conclude that native PfP2 forms homo-oligomers dominated by alpha-helical content. Multidimensional NMR investigations suggest that in the native state oligomer the C-terminal 40 residues remain flexible and exposed to solvent. In the 9 M urea denatured state the protein shows mostly non-native β structural propensity. ^15^N relaxation studies in conjunction with average area buried upon folding (AABUF) calculations suggest transient hydrophobic collapse in the denatured ensemble. This conclusion is in accord with a FT-IR study on a carefully designed polymer to study the mechanism of urea denaturation [44] but is in contrast to the results of a computational study on partially folded proteins [45]. Our results favor the “hydrophobic collapse” model of protein folding for PfP2.

## Materials and Methods

### 1. Protein expression and purification

**Figure 1:**
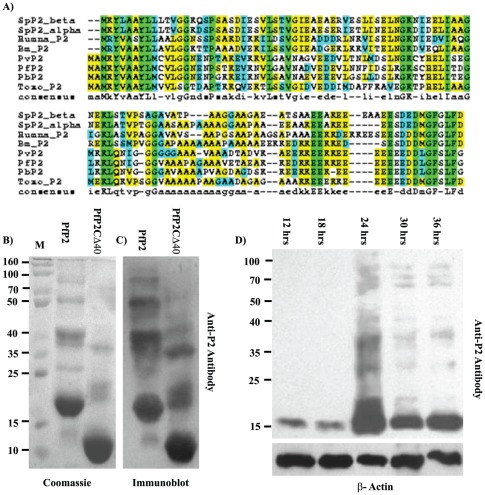
Sequence alignment, PAGE and antibody assays of P2. (A) ClustalW alignment of P2 protein across eukaryotic species. Sp: *Saccharomyces pombe* (yeast); Human: *H. sapiens*; Bm: *Bombyx mori* (silkworm); Pv: *Plasmodium vivax*; Pf: *Plasmodium falciparum*; Pb: *Plasmodium berghei*; Toxo: *Toxoplasma gondii.* The blue bar below shows the 40 amino acids deleted in the PfP2 Deletion Construct (PfP2-Del 40). The arrowhead shows the position of human P2 deletion [15]. (B) Coomassie stained SDS-PAGE of 100 µg each of recombinant PfP2 proteins. Lanes 1:PfP2; 2: PfP2-Del 40. (C) Immunoblot of the same gel as shown in Panel B probed with anti-P2 monoclonal antibody E2G12. (D) Immunoblot of 40 µg each of crude *P. falciparum* parasite protein extracts prepared from a synchronized culture at various time points post-merozoite invasion, probed with anti-P2 monoclonal E2G12. The lower panel shows a loading control of the same blot probed with anti-β actin antibody.

The cDNA encoding P2 was cloned into pPROEXHTa vector which itself codes for a 30 residue affinity tag including six histidines. Histidine-tagged P2 was overexpressed in *Escherichia coli* BL21 (DE3) cells by inducing the culture with 500 µM isopropyl-β-D-thiogalactopyranoside at 37°C for 4–5 hours. The protein was affinity purified on Ni-NTA beads (Sigma-Aldrich) and eluted with Tris buffer (pH 7.5; 20 mM) containing NaCl (150 mM), imidazole (250 mM) and DTT (5 mM). The purity of the sample was checked by western blotting using an anti-P2 monoclonal antibody (Figure 1). A similar protocol was used for a deletion construct lacking 40 residues from the C-terminus.

*Plasmodium falciparum* parasites were maintained in culture as described earlier [46]. Briefly, human blood from healthy adults with B^+^ blood group was collected in acid citrate dextrose as the anticoagulant. After removing the leukocytes, the erythrocytes were washed and resuspended in complete RPMI (cRPMI with 0.5% Albumax). Asexual stages of *P. falciparum* (3D7 strain) were synchronized using sorbitol, and maintained at 2–5% haematocrit in cRPMI at 37°C in a humidified chamber containing 5% CO_2_.

Isotope-enriched (^15^N or ^15^N/^13^C) PfP2 was prepared using M9 media containing ^15^NH_4_Cl and ^13^C glucose as the sole sources of nitrogen and carbon, respectively. The purified protein was concentrated to ∼1 mM concentration. For the urea-denatured state sample, the protein was exchanged with 100 mM MES buffer (pH 5.6) containing 150 mM NaCl, 5 mM DTT and 9 M urea. The NMR samples (containing 10% D_2_O (v/v)) were allowed to attain equilibrium before starting the experiments.

### 2. NMR spectroscopy

All the NMR experiments were recorded at 300 K on a Bruker 800 MHz spectrometer equipped with a triple resonance cryo-probe with an actively shielded Z-gradient. Series of two- dimensional and three-dimensional experiments were carried out on native and 9 M urea denatured protein. An HSQC spectrum was recorded at the end of the experiments in order to check the protein stability; we observed no change in the HSQC spectra indicating that the protein had reached equilibrium at the beginning of the experiment. Backbone H^N^ and ^15^N resonance assignments for denatured and native states were obtained using 3D HNN and HN(C)N triple resonance experiments [47], [48]. 3D HNCA, HN(CO)CA, CBCANH, CBCA(CO)NH and TOCSY-HSQC (^15^N) experiments [49], [50] provided additional checks and facilitated the assignment. ^15^N transverse relaxation rates (R_2_) were measured using CPMG delays: 17, 34, 51, 68, 85, 102, 119, 136, 153 and 187 ms. ^15^N longitudinal relaxation rates (R_1_) were measured using inversion recovery delays: 10, 30, 60, 100, 200, 300, 450, 600, 800 and 1000 ms. Steady state ^1^H-^15^N heteronuclear NOE measurements were carried out with a total 5 s interscan delay where proton saturation time was 3 s and relaxation delay was 2 s. For the experiment without proton saturation the relaxation delay was 5 s. H^N^-H^α^ coupling constants were measured from the F2 dimension of a high resolution HSQC spectrum recorded with 8192 complex t_2_ and 512 complex t_1_ points. The ^1^H chemical shift was referenced to HDO while the ^13^C and ^15^N chemical shifts were indirectly referenced to DSS [51].

### 3. Spectral density function

The spectral density at zero frequency, J(0), was calculated as described by Lefevre *et al.* [52] using the reduced spectral density approach. The reduced spectral density approach uses only three relaxation parameters (^15^N R_1_, R_2_ and {^1^H}-^15^N NOE) and assumes that at high frequencies that 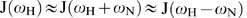. J(0) is represented as follows.
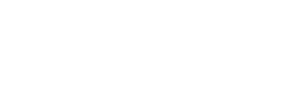
Where θ represents the 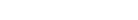


At 800 MHz the constant *c* ^2^ takes the value ∼2.25×10^9^ (rad/s)^2^ and *d* ^2^∼1.35×10^9^ (rad/s)^2^. The uncertainty in the J(0) spectral density values were calculated by standard error propagation from the experimentally estimated uncertainties in the measured relaxation parameters.

### 4. Circular dichroism

Far-UV circular dichorism (CD) spectra of the protein samples were recorded on a JASCO-J810 spectropolarimeter (Jasco, Hachioji, Japan) at 27°C, at 0 and 9 M urea concentrations using a 0.2 cm cell and a slit width of 2 nm. The protein concentrations used were 20 µM for full length PfP2 and 50 µM for deletion construct PfP2. Samples were equilibrated for at least 10–12 h before CD measurements. Each spectrum is the average of eight wavelength scans.

### 5. Average area buried upon folding

The per-residue average area buried upon folding (AABUF) was calculated using the method described by Rose *et al.,* [53] using the ExPASy website, with a nine residue moving average window.

### 6. Dynamic light scattering

Dynamic light scattering (DLS) experiments were performed on a DyanaPro-MS800 instrument (Protein Solutions Inc., Charlottesville, VA) that monitors the scattered light at 90° to the incident beam. Twenty measurements were collected, each of at least 10 s duration. Buffer solutions were filtered through 0.22 µm filters (Whatman Anodisc 13, Whatman plc, UK). Care was taken to minimize dust contamination. The ‘Regularization’ software provided by the manufacturer was used for analyzing the distribution of hydrodynamic radii of particles in solution. Standard 6 nm diameter beads and bovine serum albumin (hydrodynamic radius 3 nm) were used as standards. Experiments were performed with PfP2 concentration varying from 40 µM to 180 µM in Tris buffer using a 50 µl volume cuvette. All measurements were performed at 25°C.

### 7. Multi-angle light scattering

Multi-angle light scattering was performed on a HELEOS 8 Wyatt instrument that can monitor the scattered light at different angles. Twenty to thirty measurements were taken. Buffer solutions were filtered through a 0.22 µm filter (Whatman Anodisc 13, Whatman plc, UK). BSA was used as standard. Experiments were performed with PfP2 concentration varying from 250 µM to 400 µM.

### 8. Electrostatics calculation

In the absence of PfP2 structural information, a 3D model of the protein was generated by using I-Tasser, a platform which starts from an amino acid sequence, first generates three dimensional atomic models from multiple threading alignments and iterative structural assembly simulations [54]. Then a coordinate file was created in PQR format, which contains atomic positions of all of the atoms in addition to their charge and radius. The PQR file was generated using the Amber force field from the PDB file using the PDB2PQR [55] tool. The protein dielectric value (ε_p_) was assigned to 2 while solvent dielectric value (ε_sol_) was 78. The Electrostatic calculation was carried out at 300 K with the linearized PB equation with a solvent probe radius of 1.4 Å.

## Results and Discussion

### 1. PfP2 is oligomer in solution

Although the ribosomal P2 proteins from eukaryotic organisms are quite conserved, there appear to be distinct differences between protozoan and other P2 proteins (Figure 1A). For instance the two additional amino-terminal amino acids (Met-Ala) are present only in apicomplexan protozoans but not in other eukaryotic organisms. The serine residue present at the 12th amino acid position from the carboxy-terminal end, which undergoes phosphorylation and plays a regulatory role in both humans and yeast, is missing in protozoan P2; instead a serine residue is found at the 4th last position, absent in higher eukaryotes (Figure 1A). Recombinant PfP2 protein undergoes self-association at concentrations greater than 20 µM and does not dissociate even in the presence of SDS-detergent and DTT treatment (Figure 1B, C). In the case of human P2 protein, deletion of 46 residues from the carboxy-terminal domain was shown to abolish the propensity to form higher molecular weight oligomers [15]. The truncation in the human P2 protein was done just beyond the two conserved GG residues (Figure 1A). A PfP2 deletion construct with 40 amino acids missing from the C-terminal end, which contained two more residues compared to that in the human P2 deletion construct (Figure 1A) continued to form homo-oligomers (Figure 1B,C).

*Plasmodial* P2 protein appears to play a distinct non-ribosomal novel role in the G0 to G1 transition of parasite cell division in infected erythrocytes. This is demonstrated through the blockade of cell division in the presence of a panel of monoclonal antibodies specific to *Plasmodium* P2 protein (Das *et al.*, ms communicated). Of the three P-proteins, P2 protein alone translocates to the infected RBC surface for 6–8 hours prior to nuclear division, and this translocation is concomitant with extensive oligomerization of the parasite P2 protein. During this sub-stage, an exclusive presence of a P2 homotetramer is detected on the infected RBC membrane (Das *et al.*, ms communicated).

In the erythrocyte, *Plasmodium falciparum* develops through the ring stages up to about 18 hours post merozoite invasion (PMI), followed by the growing trophozoite stages (18–36 hrs PMI). The PfP2 protein exists as a monomer during the ring stages, but at the onset of the cell division (around 24 hrs PMI), exhibits massive detergent resistant oligomerization (Figure 1D). The amount of P2 protein is also found to oscillate with the highest amount of P2 protein being formed at 24 hrs PMI. Within the erythrocytic development cycle of 48 hrs in synchronized *Plasmodium falciparum* cells, the high concentration of PfP2 protein and its extensive oligomerization mainly at 24 hrs PMI indicates a definitive role for PfP2 oligomerization, the precise nature of which is yet to be elucidated.

**Figure 2:**
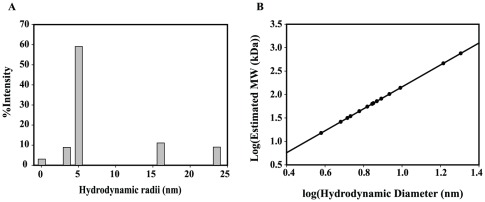
Dynamic light scattering analysis. (A) Histogram of distribution of hydrodynamic radii obtained from regularization analysis of data from dynamic light scattering for 300 µM P2 in 20 mM Tris buffer, 150 mM NaCl at pH 7.5; average R_h_ 5.0. (B) Plots of hydrodynamic diameter for 14 different proteins from [80].

Light scattering experiments were performed to estimate the molecular mass of the dominant PfP2 species in solution. Dynamic light scattering (DLS) experiments yield the hydrodynamic radius of different species present in the solution reflecting the association state of the protein. DLS measurements were performed on PfP2 at 25°C, pH 7.5 for different protein concentrations in the range 40 to 180 µM. In all the cases the results are shown for freshly prepared samples; typical data is shown in Figure 2. The results suggest that there is one major species with a hydrodynamic radius of ∼5 nm accompanied by weaker bands at ∼2–3 nm, 16 nm and 25 nm. We observed that on storage population of higher molecular weight species (i.e. with radii ∼16 nm and ∼25 nm) increases. By way of control Figure 2B presents a plot of the average hydrodynamic diameters for many different proteins with known molecular mass (proteins are listed in Supplementary Table S1) and a correlation plot of estimated and observed molecular weight is shown in Supplementary Figure S2. This graph demonstrates a linear relationship between the predicted hydrodynamic diameter (D_h_) and molecular weight that we fit to the following equation:
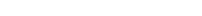
From this plot, the highly populated species D_h_ (10.06 nm) of PfP2 corresponds to an average molecular weight of ∼138 kDa which indicates that PfP2 forms a multimeric oligomer; since the monomer molecular weight is 15.5 kDa, the observed size indicates association of ∼8–9 monomer units in solution. *In vivo* results (see Figure 1D), however, seem to indicate that all types of PfP2 species are present but only the homotetramer is found to associate with the infected RBC membrane. It could be that under *in vitro* conditions two such tetramers may self-associate further to form an octameric species. The light scattering band at D_h_ = 4 nm fits with the putative PfP2 monomer and those at larger hydrodynamic radii reflect higher order states of association. The formation of PfP2 oligomers was also confirmed by multi-angle light scattering. At 250 to 400 µM; the molecular weight observed for a freshly prepared sample of PfP2 is 117 kDa with n ∼7–8 where n represents size of the aggregates.

### 2. PfP2 is largely helical in nature

**Figure 3:**
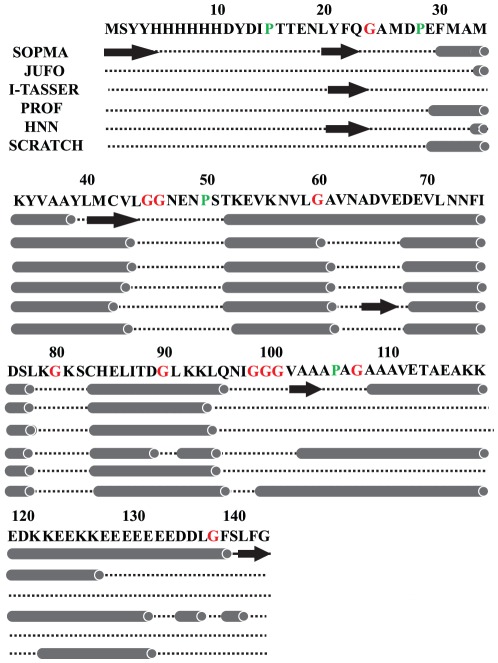
Secondary structure predictions. Summary of structure prediction details of PfP2 using six different programs. Cylinders show α-helical regions, arrows show β sheet and lines show random coils.

The intrinsic secondary structural preferences for PfP2 were predicted using several well-validated secondary structure prediction algorithms [54], [56], [57], [58], [59], [60]. The results are shown in Figure 3. We observed that the predictions by the six algorithms were very similar, establishing that the predictions are reasonably reliable. Four helices are consistently predicted for the stretches Met33-Tyr39, Thr52-Gly60, Asp68-Leu78 and Cys83-Leu94.

**Figure 4:**
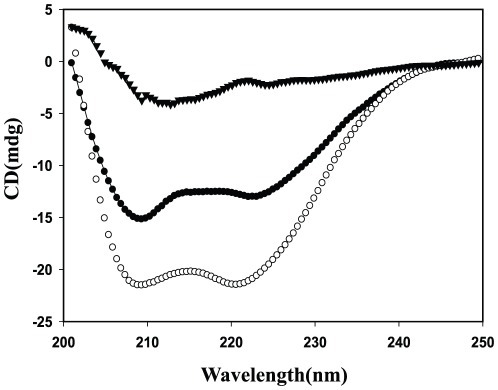
CD spectra of PfP2. Far- UV CD spectrum of Full length P2 (filled circles ·), Deletion construct of P2 (open circles o) at pH 7.5 and 27°C and in 9 M urea of P2 at pH 6.5 and 27°C (star mark).

We recorded far UV CD spectra of PfP2 to estimate the secondary structure content of the protein. For human P2 it has been reported that deletion of the last 46 residues increases the stability of the dimeric protein and reduces higher order self-association [15]. Therefore, we investigated both full length PfP2 as well as a corresponding deletion construct; the results are shown in Figure 4. After normalizing CD intensity to the protein concentration, the helical content in both the cases is found to be 30–35%. As a reference we also recorded the CD spectrum of denatured PfP2 in 9 M urea. The absence of significant CD signal under these conditions indicates that the helical secondary structure is almost entirely lost.

### 3. The conserved C-terminus of PfP2 does not participate in self-association

**Figure 5:**
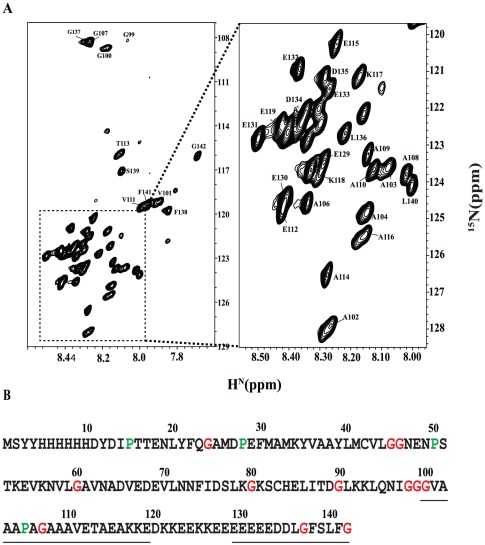
NMR assignments in native PfP2. (A) 2D ^1^H,^15^N HSQC spectrum of Native PfP2 in aqueous solution at pH 6.5 and 27°C. Residue specific assignment for each peak is marked on the spectrum (B). Assigned residues are underlined on the sequence.

**Figure 6:**
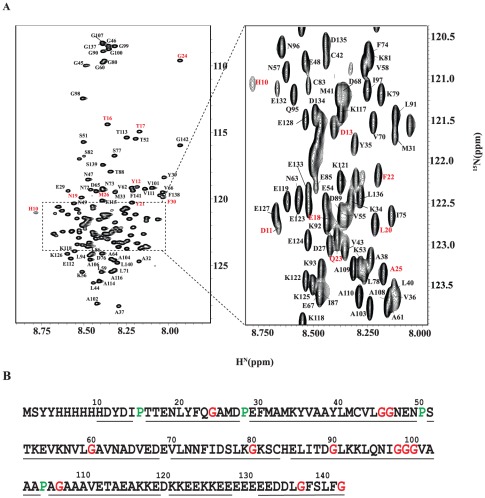
NMR assignments in urea denatured PfP2. (A) 2D ^1^H,^15^N HSQC spectrum of PfP2 in 9 M urea at pH 5.6 and 27°C. Residue specific assignment for each peak is marked on the spectrum (B) Summary of sequential assignment. In A, Peaks from the vector are marked in red. Out of 112 protein residues (138 minus 26 which derive from the vector) excluding the two proline residues, 104 have been sequence-specifically assigned; the Vector peaks have also been assigned. Six peaks could not be assigned because of not availability of sequential connectivities. The spectra contained some additional peaks of less intensity which also showed some sequential connectivity. These are more likely due to existence of some other conformation in slow exchange. Assignment is submitted in BMRB under the accession code 17616.

The ^1^H-^15^N HSQC spectrum of native PfP2 protein shows only 43 backbone amide NH cross peaks compared to an expected tally for a monomeric chain of 138 peaks (Figure 5). The spectrum was unchanged over the concentration range 300–800 µM. To check that the sample contained full length protein we denatured the sample in 9 M urea and re-recorded the HSQC spectrum (Figure 6). This spectrum shows the full complement of cross peaks predicted from the protein sequence. Moreover all the cross peaks could be sequence-specifically assigned in the denatured state. Illustrative sequential walks through the segments G137-L140 in the native state and L44-E48 in the urea denatured state of PfP2 are shown in Supplementary Figure S1 using the F_1_–F_3_ planes from the HNN NMR spectrum.

The fact that there are only a small number of peaks that belong to the C-terminus of the polypeptide chain in the native state spectrum can have several explanations. This could be either because of formation of stable high molecular weight aggregates involving a section of the polypeptide chain while the rest of the chain is freely mobile, or because of line broadening due to intermediate exchange in the solution as a result of self-association equilibria and/or conformational variation in solution. The light scattering experiments indicate that PfP2 forms a high molecular weight oligomer in solution. The fact that dilution did not affect the HSQC spectrum indicates that there is apparently no equilibrium between the monomer and the multimer and that even if present, the balance is highly in favor of the multimer state. Thus we conclude that a C-terminal stretch of ∼40 residues is free and flexible in the oligomer and does not participate in aggregation, and that the rest of the chain is not seen in the spectrum because of its involvement in chain self-association. After exchange of the native state sample in deuterium oxide the C-terminal residue cross peaks disappeared in less than 5 minutes indicating that the C-terminal amide NH groups are well exposed to the solvent. Removal of the last 40 residues from the C-terminus of PfP2 led to an almost blank HSQC spectrum (data not shown) indicating that the truncation does not prevent aggregation, as was observed in the case of human P2 [15].

### 4. Insights into folding and self-association

In order to gain further structural and dynamic insight into PfP2 we decided to further characterize the denatured state. Denatured states of proteins often possess residual structure and a pattern of dynamics which reflect upon folding initiation sites for the native protein. Thus we have extensively characterized the denatured state of PfP2 whose spectrum contains the signature of the entire protein.

#### 4.1 Structural preferences in the denatured state

Deviations of observed NMR chemical shifts from random coil values give an indication of secondary structure present in proteins. The random coil values are derived from the spectra of short peptides having 5–6 residues anticipated to have no residual structural order. There is more than one set of random coil shift values reported in the literature which differ in the corresponding measurement conditions: one due to Wishart and Sykes [51], [61] and another by Schwarzinger *et al.* [62]. The former was recorded in pH 5 and 1 M urea while the latter used pH 2.3 and 8 M urea. We used the sequence-corrected random coil values reported by Schwarzinger *et al*. [62] for the assessment of secondary chemical shifts for denatured PfP2. Of the various secondary chemical shifts (Δδ), those of H^α^, ^13^C^α^ and ^13^CO are the most diagnostic of the local structural propensity of the polypeptide chain [63], [64]. Thus if some residues show positive (downfield) H^α^ and negative (upfield) ^13^C^α^ and ^13^CO chemical shift deviations from random coil values (also called secondary shifts), then those residues are taken to have a preference for the β-domain of Ramachandran space. Residues with α-helical propensity show the opposite secondary shift pattern.

**Figure 7:**
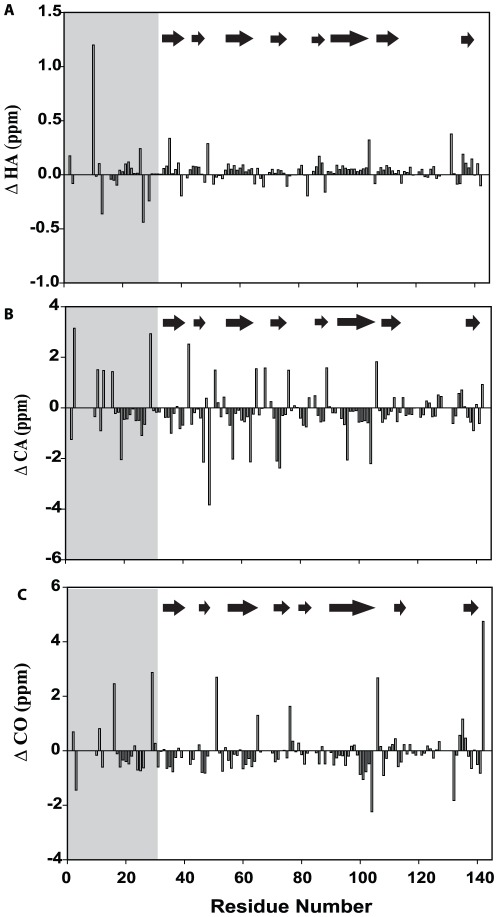
NMR secondary chemical shifts. (A) ΔH^α^ secondary chemical shift (B) ΔC^α^ secondary chemical shift for (C) ΔCO secondary chemical shift of 9 M urea denatured state of PfP2 at pH 5.6 and 27°C. The secondary structural propensities are indicated in each box; arrow indicates β-propensity. Shaded grey region represents a stretch that is coming from the vector and is not part of PfP2.

We used H^α^, ^13^C^α^ and ^13^CO chemical shifts to characterize the residual structure present in 9 M urea denatured PfP2. The overall pattern of secondary shifts (Figure 7) suggests that the denatured chain is not a pure random coil. Though the secondary shifts are small they indicate an overall bias for β structural preference. Considering that the protein is thought to be largely helical in the native state, this result suggests that in the urea-denatured state the protein has highly non-native conformational preferences.

We measured ^3^J(H^N^-H^α^) coupling constants for all the residues in PfP2. These data give an indication about backbone torsion angles. For a regular helical structure ^3^J(H^N^-H^α^) is 3–5 Hz, but 8–11 Hz for β-structure [64], and typically 6–8 Hz for random coils. It has been seen that the random coil ^3^J(H^N^-H^α^) value for a given residue is influenced by the nearest neighbor residues along the sequence: two sets of standard sequence specific J-corrections values have been reported in the literature, depending upon whether the N-terminal residue belongs to one of two subsets of amino acids [65]. For PfP2 we calculated the deviation of the observed coupling constant from the predicted sequence-dependent random coil value, (J_obs_-J_rc_), which gives another insight into the secondary structural propensity in the denatured state. A negative secondary coupling constant indicates helical propensity and a positive value indicates β structural propensity [65]. The combined use of secondary coupling constants with secondary chemical shifts provides an enhanced indication about the local secondary structure propensity of the chain. It also enables distinguishing between β and PP_II_ structures [65]. For β structure both the H^α^ secondary chemical shifts and secondary coupling constant would be positive, whereas for a PP_II_ helix the secondary coupling constant would be negative.

**Figure 8:**
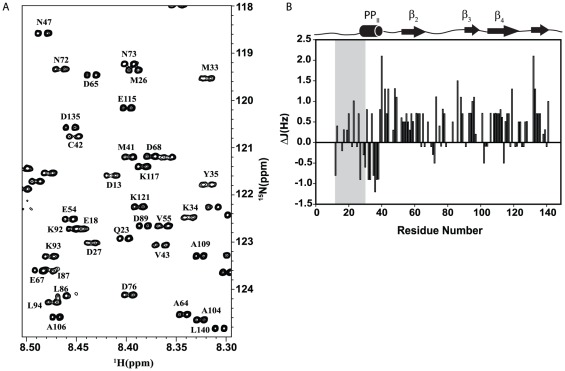
Coupling constant measurements in denatured PfP2. (A) Selected region from a high resolution 2D ^1^H,^15^N spectrum of PfP2 in 9 M urea at pH 5.6 and 27°C. The splitting in the well resolved peaks is used to measure the ^3^J(^H^N-H^α^) coupling constants (B) Secondary coupling constants are plotted against sequence for P2. The secondary structural propensities are indicated on top of the box; arrow indicate β-propensity, cylinder denotes PP^II^ helix. Shaded grey region represents a stretch that is coming from the vector and is not part of PfP2.

The measured values of the secondary coupling constants from the high resolution HSQC spectrum of PfP2 (Figure 8) show that the deviations for most of the residues are smaller than ∼1.0 Hz. We note that the precision of secondary coupling constant estimation is ∼0.5 Hz for the positive values and slightly worse but <∼1.0 Hz for negative deviations; this is because negative deviations occur when coupling constants themselves are small. In Figure 8 most of the residues show positive secondary chemical shifts and positive secondary coupling constants indicating that they mainly populate β structure. We also find a small stretch, Phe30-Ala38, which displays negative secondary coupling constants but positive H^α^ secondary chemical shifts, thus indicating a PP_II_ structure. A residue-wise assessment of β or PP_II_ preferences from combined use of secondary coupling constants and secondary chemical shifts is given in Figure 8.

#### 4.2 Motional characteristics and folding initiation sites

**Figure 9:**
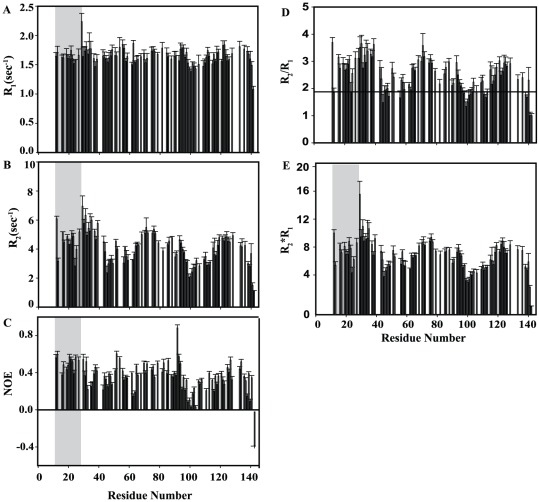
NMR relaxation measurements. ^15^N R_1_, R_2_ and {^1^H}-^15^N heteronuclear NOE Relaxation parameters recorded at 800 MHz versus the residue numbers for 9 M urea denatured state of PfP2 in 100 mM MES buffer at pH 5.6 and 27°C (a) ^15^N R_1_ (longitudinal relaxation rate) (b) ^15^N R_2_ (transverse relaxation rate) (c) {^1^H}-^15^N heteronuclear NOE (d) R_2_/R_1_; the line indicates the floor value for R2/R1 (1.88) (e) R_2_*R_1_ versus residue numbers. The black bar indicates regions with reduced flexibility [marked as A (Met31-Tyr39), B (Ala64-Lys79), C (Glu85-Thr88), and D (Lys118-Lys136)] in the polypeptide sequence and gray bar indicates regions with higher flexibility: D (Leu44-Asn63) and E (Gln95-Gly117). Shaded grey region represents a stretch that is coming from the vector and is not part of PfP2.

NMR relaxation measurements provide a powerful tool for investigating dynamic properties of proteins over a wide range of time scales [66], [67], [68]. Molecular motions of unfolded and partially folded proteins are highly heterogeneous and dynamic processes associated with global and internal motions occur on multiple time scales. Backbone dynamics and overall structural fluctuations for the urea-denatured PfP2 were assessed with ^15^N R_1_ (longitudinal relaxation rate constant), ^15^N R_2_ (transverse relaxation rate constant), and {^1^H}-^15^N heteronuclear NOE measurements all recorded at 800 MHz (Figure 9). R_1_ and {^1^H}-^15^N heteronuclear NOE values are most sensitive to fast motions (ns-ps time scale), whereas the R_2_ value is sensitive to low frequency motions (ms-µs time scale) and includes contributions from intermediate exchange processes. For a denatured state of a protein chain where conformational states and correlation times are expected to be highly heterogeneous, these relaxation rates are typically interpreted in a qualitative fashion to suggest motional trends along the chain.

The relaxation rates were determined for 92–95 non-overlapping ^N^H cross peaks of PfP2 out of a total of 126 assigned residues of which 112 residues are from the protein proper and the remaining are part of the affinity purification tag. The derived relaxation rate constant data is presented in Supplementary Table S2. In the denatured state considerable variation in the R_2_ values is seen along the chain. The values range from 1.07 to 6.07 s^−1^ (mean R_2_ value: 4.07±0.26 s^−1^). This variation can arise either because of local structural and motional variations or because of different degrees of conformational exchange contribution to transverse relaxation. However considerable non-randomness of the observed R_2_ values suggests the presence of significant segmental conformational dynamics in the urea denatured state of PfP2. Because of this conformational heterogeneity almost every residue has a contribution from conformational exchange (R_ex_) to its transverse relaxation rate.
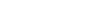
Where R_2_,int is the intrinsic transverse relaxation rate associated mainly with the local overall reorientational motion (tumbling) of the NH bond vector.

In Figure 9, there are distinct clusters of residues which show R_2_ values higher than the chain average: Met31-Tyr39: 5.45 s^−1^; Ala64-Lys79: 4.84 s^−1^; Glu85-Thr88: 4.54 s^−1^; and Lys118-Lys136: 4.54 s^−1^. These regions may be considered to be exhibiting slow segmental motions. Broadly speaking these regions coincide with those identified as having a β-structure propensity on the basis of secondary chemical shift analysis. Therefore, one can speculate that this slow dynamics might be because of β to α structural transitions occurring on ms-µs time scale in this region, which has largely β (non-native) propensity in the urea denatured state but is predicted to have α-helical structure in the native state. We also observed regions which show relatively small R_2_ values. Residues which show R_2_ values lower than average signify greater flexibility. These segments of PfP2 include: Leu44-Asn63 (mean R_2_ 3.42 s^−1^), Gln95-Gly117 (3.09 s^−1^). Of these two segments, Gln95-Gly117 is predicted by the pattern of secondary chemical shifts to have random coil structure and thus it may act as a hinge for surrounding segmental motions.

The R_1_ value is sensitive to both low and high frequency motions on the ns to ps time scale. For urea-denatured PfP2 the R_1_ values range from 1.07 to 2.23 s^−1^ (mean R_1_ 1.64±0.07 s^−1^). The C-terminal residues of the polypeptide show particularly small R_1_ values indicating much faster motions in this region. Elsewhere there is some variation along the chain. For example a large value of R_1_ is observed for stretches: Met31-Tyr35, mean R_1_ 1.75 s^−1^; Glu48-Gln57, 1.73 s^−1^; Ile75-Glu85, 1.72 s^−1^; Leu94-Asn96, 1.78 s^−1^; and Glu133-Phe139, 1.74 s^−1^. In the stretch Gly99-Val111 the R_1_ values are lower than the overall average value which likely indicates faster motion for this region.

The {^1^H}-^15^N steady state NOE values provide information about ps timescale motions. In the denatured state of PfP2 the heteronuclear NOE values (Figure 9) range from −0.36 to +0.88 (mean NOE 0.36±0.02). It is important to note here that all the relaxation parameters are smaller for the C-terminal residues indicating more conformational flexibility in this region while the N-terminal region appears relatively more ordered. It can be seen that there are two clusters of residues, Val43-Asn49 and Gln95-Val111, with mean NOE values of 0.30 and 0.20 respectively, lower than the global average value indicating large amplitude picosecond timescale motion.

#### 4.3 Conformational exchange in the denatured state

As discussed above, the R_2_ values have the potential to throw light on conformational exchange processes occurring along the chain; relatively large R_2_ values indicate the presence of exchange at those sites. Both R_1_ and R_2_ relaxation rates include contributions form dipolar and chemical shift anisotropy (CSA) interactions. However conformational exchange on ms-us time scale contributes only to R_2_. Thus, for particular NH groups in a putative globular molecule a significant elevation in the R_2_/R_1_ ratio [69], [70] can be taken to indicate a contribution from conformational exchange and non-uniformity in R_2_/R_1_ has been used to demonstrate the presence of nonrandom structures in urea-denatured states of various proteins [71], [72], [73], [74], [75], [76]. In the present case, considering the regions of the chain which are identified to have a more random coil conformations and low R_2_ values indicating maximal flexibility (Gln95-Thr113) a “floor value” for R_2_/R_1_ to represent a truly unfolded state devoid of slow conformational exchange is estimated at 1.88; any R_2_/R_1_ value above this floor can then be attributed to conformational exchange. On this basis the R_2_/R_1_ ratio is high for the regions Met31-Tyr39, Ala64-Lys79, Glu85-Thr88 and Lys118-Glu128. Interestingly, these regions, except Lys118-Lys136, coincide well with those where secondary structures have been predicted in the native state and thus it may be conceived that these three regions could be sites of folding initiation. While the secondary shifts indicate that these regions have mostly propensity for β-structure in the urea-denatured state (Figures 7 and 8), the secondary structure prediction algorithms indicate helical structure in the native state. Thus it is possible to imagine exchange between non-native β- and native helical structure for these regions. To further exclude the possibility of high R_2_/R_1_ ratios due to low R_1_ values, rather than due to large R_2_, the R_2_R_1_ product analysis was also calculated which is shown in Figure 9. From all of these, it may be concluded that the residues in these region Met31-Tyr39, Ala64-Lys79 and Glu85-Thr88 slow segmental motions in the urea-denatured state of PfP2.

### 5. Hydrophobic collapse initiates folding

Further insights into polypeptide chain order-disorder dynamics can be obtained by analyzing the correlation between R_2_ values or the zero frequency spectral density, J(0), which reflects ms-µs time scale internal motions and conformational exchange, and average area buried upon folding (AABUF) [53]. AABUF provides a set of empirical parameters developed by Rose *et al.* [53] to indicate the accessible surface area lost when a residue becomes buried upon folding. The AABUF output is proportional to the hydrophobic contribution of a residue to the conformational free energy of the protein and has been shown to correlate well with sequence dependent dynamic variations due to hydrophobic cluster formation in denatured states [71].

**Figure 10:**
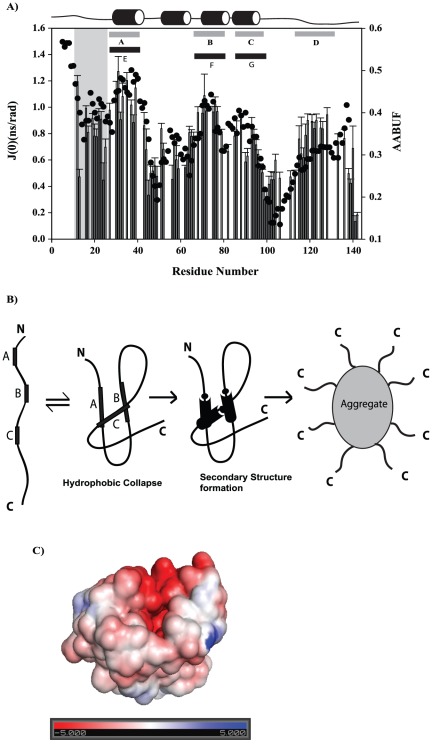
Insights into folding and self-association. (A) Plots of calculated J(0) values at 800 MHz and AABUF (average area buried upon folding) for 9 M urea denatured state of PfP2. The AABUF were calculated with the expasy tool protscale [https://www.us.expasy.org/tools/protscale.html]. The predicted secondary structure is shown on top (see Figure 3). The regions with increased AABUF values have been marked with black bars and those with reduced flexibility have been marked with grey bars. Shaded grey region represents a stretch that is coming from the vector and is not part of PfP2 (B) Schematic Representation of Hydrophobic collapse model of folding and subsequent association of PfP2. (C) The electrostatic potential (isocontour value ±10 KT/e) surface of PfP2 calculated using a theoretically generated 3D structure of the protein with surface amino acid charges are depicted in red (negative charge) and blue (positive charge). Neutral elements are depicted in white color. This clearly indicates that the surface is highly hydrophobic in nature.

Thus, the variations in R_2_ can result from differences in short range interactions because of different local structural preferences and from conformational exchange. A correlation between J(0) and AABUF can be a useful identifier of regions involved in such exchange. [77], [78]. The calculated AABUF values for PfP2 along with experimentally derived J(0) values obtained from the relaxation parameters are shown in Figure 10. Interestingly, there is a close parallel between the AABUF and J(0) plots; regions of high AABUF coincide with regions of high J(0). Three distinct regions of combined high AABUF and J(0) values can be identified: Met31-Leu44, Val70-Leu78 and Ser82-Leu91. In PfP2, which is prone to self-association, the exchange can be either between open and compact structures created by hydrophobic collapse (case 1) or between open monomeric and self-associated species, the association occurring via hydrophobic interactions (case 2). Case 1 is in accordance with a recent report that urea can, in fact, favor hydrophobic collapse of a polymer chain representing the backbone of a protein [44]. In this case the hydrophobic patches listed above indicate folding initiation sites in the monomer when appropriate conditions are provided by dilution of the denaturant. In all these regions α-helical secondary structures have been predicted in the native state but the experimental data suggest tendency to form β-structure in the denatured state. In the latter case, the hydrophobic patches identify the association initiation sites along the chain. In fact both of these mechanisms can actually be occurring in the denatured ensemble in solution. We will explicitly address the issue of exchange between open monomeric and self-associated species by performing, in future, extensive relaxation measurements in the denatured state at different protein concentrations.

To explore further case 1 described above, where the monomer folds before the onset of self-association, we calculated the electrostatic surface potentials for a 3D model generated by I-Tasser [54] using the adaptive Poisson Boltzmann solver (APBS) tools [79] in PYMOL (Figure 10C). Considering all the residues for which the accessible surface area (calculated using NOC http://noch.sourceforge.net/) exposed is more than 40% and from among them the residues that are hydrophobic in nature, we estimate the hydrophobic surface to be approximately 23% of the total surface area. This is not contiguous, however, but is distributed in several patches. These would promote self-association from different sites on the surface. Following this model, a possible scheme to describe the overall folding and self-association of PfP2 is imagined in Figure 10B, wherein the flexible and highly charged C-terminal segments of the individual molecules in the oligomer are shown to be projected in a disordered fashion into the solution while the N-terminus is buried inside the particle core. We do not know at this stage the structural details of this core and further investigation using additional methods will be required to characterize these aspects.

### Conclusions

In *Plasmodium* species, SDS-resistant P2 homo-oligomers are detected at certain erythrocytic stages of development, and these appear to be involved in extra-ribosomal functions in the parasites. Therefore it is important to understand the self-associative properties of the *Plasmodial* P2 protein. In this paper we have made some progress in deciphering the general characteristics of PfP2, although the atomic level structure is still far from clear. Our data suggest that the PfP2 has an intrinsic propensity to oligomerize in solution, and the structure cannot be probed in detail in the native state. Insights into folding and self-association have been derived by investigating the denatured state of the protein where it is possible to analyse the full signature of the protein by NMR. Our data indicates that the urea-denatured state of PfP2 mostly has structural propensities in the β-region of the Ramachandran map over the majority of the chain length, though one stretch shows PP_II_ behavior. The polypeptide chain has significant conformational restriction and shows sequence dependent variation of its dynamic properties. From the combined estimated of local structural propensity, the motional characteristics in the denatured state and AABUF calculations we infer that stretches Met31-Tyr39, Ala64-Lys79 and Glu85-Thr88 have hydrophobic character and could be the initiation sites for folding, if folding precedes self-association, or sites of intermolecular association if this precedes or goes in parallel with folding. It is possible that both mechanisms may be operating in solution. To gain further insights into the association process, in case folding precedes self-association, we calculated the electrostatic potential surface of the protein using predicted 3D model of PfP2. This indicated that the surface of the folded protein may have patches of hydrophobic character, which provide sites for intermolecular association in aqueous solution.

## Supporting Information

Figure S1 **Illustrative sequential assignments.** (A) Sequential walk through the F_1_–F_3_ planes of HNN spectrum of P2 in 9 M urea at pH 5.6 and 27°C. Sequential connectivities are shown for L44 to E48 stretch. F_2_(^15^N) values are shown at the top for each strip (B) Sequential walk through the F_1_–F_3_ planes of HNN spectrum of Native P2 in aqueous at pH 6.5 and 27°C. Sequential connectivities are shown for G137 to L140 stretch. F_2_(^15^N) values are shown at the top for each strip. Black and red shows positive and negative peaks. Distinct Gly serves as check point in the sequential assignment.
(EPS)

Figure S2 **Correlation of DLS-estimated** ***vs.*** **actual molecular weight.** Using 14 different proteins (Supplementary Table S1), the estimated molecular weights from DLS measurements have been plotted against the actual molecular weights. The correlation coefficient is found to be 0.99.
(EPS)

Table S1 **The average hydrodynamic diameter and molecular weight obtained from DLS measurements on proteins with sizes ranging from 15 kDa to 750 kDa.** Estimated molecular weight is also shown.
(DOC)

Table S2 **Relaxation data of 9 M urea denatured state of PfP2.**
(DOC)
