# Supplementary material for: NMR Insights into Folding and Self-Association of Plasmodium falciparum P2
Source: PLoS One. 2012 May 2;7(5):e36279. doi: 10.1371/journal.pone.0036279 (PMC3342256; doi:10.1371/journal.pone.0036279)
Supplement: Table S1 — The average hydrodynamic diameter and molecular weight obtained from DLS measurements on proteins with sizes ranging from 15 kDa to 750 kDa. Estimated molecular weight is also shown. (DOC) [file pone.0036279.s003.doc]

Supplementary Table S1:

| Protein | ~Hydrodynamic Dia (nm) | Known Molecular weight (kDa) | Estimated Molecular Weight (kDa) | % error |
| --- | --- | --- | --- | --- |
| Lysozyme | 3.8 | 14.7 | 15.1 | ~2.7 |
| Chymotrypsinogen | 4.8 | 25 | 26.1 | ~4.4 |
| Carbonic Anhydrase | 5.2 | 29 | 31.5 | ~8.6 |
| Human Insulin | 5.4 | 34.2 | 34.4 | ~0.6 |
| Ovalbumin | 6 | 43 | 44 | ~2.3 |
| Hexokinase subunit | 6.6 | 51 | 55 | ~7.8 |
| Hemoglobin | 7 | 65 | 63 | 3.1 |
| Bovine Serum Albumin | 7.1 | 67 | 65.3 | 2.5 |
| Horse Alcohol Dehydrogenase | 7.4 | 80 | 71.9 | 10.1 |
| Amyloglucosidase | 7.8 | 90 | 81.3 | 17.9 |
| Hexokinase | 8.6 | 102 | 102.2 | ~0.2 |
| Yeast Alcohol Dehydrogenase | 9.8 | 150 | 138.7 | 7.5 |
| Apoferritin | 16.4 | 443 | 462.2 | ~4.4 |
| Thyroglobulin | 20.2 | 669 | 753.5 | ~12.6 |
