# Supplementary material for: NMR Insights into Folding and Self-Association of Plasmodium falciparum P2
Source: PLoS One. 2012 May 2;7(5):e36279. doi: 10.1371/journal.pone.0036279 (PMC3342256; doi:10.1371/journal.pone.0036279)
Supplement: Table S2 — Relaxation data of 9 M urea denatured state of PfP2. (DOC) [file pone.0036279.s004.doc]

Supplementary Table S2:

| Residue | R_1_(s^-1^) | R_2_(s^-1^) | NOE |
| --- | --- | --- | --- |
| Y12 | 1.6447 | 6.0790 | 0.5591 |
| D13 | 1.7103 | 3.1546 | 0.5953 |
| T16 | 1.6231 | 5.1230 | 0.3742 |
| T17 | 1.6173 | 4.4385 | 0.4853 |
|  |  |  |  |
| N19 | 1.6625 | 4.8662 | 0.4326 |
| L20 | 1.6132 | 4.3122 | 0.4792 |
| Y21 | 1.6000 | 4.3422 | 0.5705 |
| F22 | 1.7458 | 5.0556 | 0.5422 |
| Q23 | 1.6054 | 4.8591 | 0.5173 |
| G24 | 1.5186 | 2.8361 | 0.3896 |
| A25 | 1.5593 | 3.9857 | 0.5614 |
|  |  |  |  |
| D27 | 1.6617 | 5.1787 | 0.5324 |
| E29 | 2.2361 | 6.9541 |  |
| F30 | 1.6393 | 5.7637 | 0.5432 |
| M31 | 1.7334 | 6.3251 | 0.3673 |
| A32 | 1.7950 | 4.9407 | 0.5191 |
| M33 | 1.6821 | 5.4259 | 0.2190 |
| K34 | 1.7687 | 5.2356 |  |
| Y35 | 1.7727 | 5.9916 | 0.2523 |
|  |  |  | 0.2881 |
| A37 | 1.6038 | 5.2247 | 0.3870 |
| A38 | 1.4691 | 4.6533 | 0.4587 |
| Y39 | 1.5944 | 5.7737 | 0.4186 |
|  |  |  |  |
|  |  |  |  |
| V43 | 1.6477 | 4.4964 | 0.2139 |
| L44 | 1.6179 | 3.8110 | 0.3476 |
| G45 | 1.5931 | 2.3646 | 0.3276 |
| G46 | 1.5468 | 2.9780 | 0.2491 |
| N47 | 1.6171 | 3.1686 | 0.3874 |
| E48 | 1.6543 | 3.3289 | 0.3575 |
| N49 | 1.7569 | 3.0048 | 0.2705 |
| S51 | 1.6595 | 4.5331 | 0.4202 |
| T52 | 1.6513 | 3.9968 | 0.6028 |
|  |  |  |  |
| E54 | 1.7989 |  | 0.5517 |
|  |  |  |  |
| K56 | 1.8295 | 3.0331 | 0.4186 |
| N57 | 1.7828 | 4.1085 | 0.3218 |
| V58 | 1.5562 | 3.4698 | 0.3556 |
| L59 | 1.6399 | 3.2196 | 0.3389 |
|  |  |  |  |
|  |  |  |  |
| V62 | 1.4995 | 3.2165 | 0.3921 |
| N63 | 1.8646 | 3.6403 | 0.1455 |
| A64 | 1.5370 | 4.2123 | 0.1879 |
| D65 | 1.5701 | 4.2790 | 0.4722 |
| V66 | 1.5954 | 4.2391 | 0.3717 |
|  |  |  |  |
| D68 | 1.6351 | 4.9950 | 0.3798 |
| V70 | 1.6935 | 5.0813 | 0.5005 |
| L71 | 1.5333 | 5.4885 | 0.3959 |
| N72 | 1.6103 | 5.1073 | 0.4946 |
|  |  |  |  |
|  |  |  |  |
| I75 | 1.7322 | 5.0968 | 0.4288 |
| D76 | 1.7440 | 5.3706 | 0.4629 |
| S77 | 1.7271 | 5.0556 | 0.3502 |
|  |  |  |  |
| K79 | 1.6779 | 4.4189 | 0.5035 |
|  |  |  |  |
|  |  |  |  |
| S82 | 1.7835 | 3.8700 | 0.4062 |
|  |  |  | 0.5055 |
| E85 | 1.6929 | 4.2974 | 0.3887 |
| L86 | 1.6359 | 4.5537 | 0.5289 |
|  |  |  |  |
| T88 | 1.5964 | 4.7619 | 0.3542 |
|  |  |  | 0.3526 |
| G90 | 1.6410 | 3.4626 | 0.3902 |
| L91 | 1.6611 | 3.6456 | 0.3718 |
|  |  |  | 0.8819 |
| K93 | 1.5672 | 4.6318 | 0.5728 |
| L94 | 1.7658 | 4.4170 | 0.4960 |
| Q95 | 1.7899 | 3.9714 | 0.2177 |
| N96 | 1.7889 | 3.7538 | 0.3479 |
| I97 | 1.6292 | 3.1075 | 0.2099 |
| G98 | 1.7126 | 3.1192 | 0.3169 |
| G99 | 1.5291 | 2.0471 | 0.0806 |
| G100 | 1.4243 | 2.0982 | 0.1055 |
| V101 | 1.3810 | 2.4178 | 0.0249 |
| A102 | 1.5115 | 2.7130 | 0.1719 |
| A103 | 1.4605 | 2.7255 | 0.2310 |
| A104 | 1.4304 | 3.2000 | 0.0317 |
| A106 | 1.5067 | 2.8409 | 0.3068 |
|  |  |  | 0.2765 |
|  |  |  | 0.3271 |
| A109 | 1.4732 | 3.2680 |  |
| A110 | 1.5326 | 3.4916 |  |
| V111 | 1.6300 | 2.8827 | 0.2071 |
| E112 | 1.7419 | 2.9078 |  |
| T113 | 1.6439 | 3.0998 | 0.3956 |
|  |  |  |  |
| E115 | 1.5147 | 4.0766 | 0.3184 |
| A116 | 1.6764 | 3.6193 | 0.1988 |
| K117 | 1.5550 | 3.5411 | 0.3632 |
| K118 | 1.7036 | 4.2571 | 0.2726 |
| E119 | 1.7179 | 4.7870 | 0.3638 |
| K121 | 1.5555 | 4.6926 | 0.3945 |
| K122 | 1.8305 | 4.5620 | 0.3226 |
| E123 | 1.8386 | 4.7985 | 0.2756 |
| E124 | 1.6493 | 4.8450 |  |
| K125 | 1.5676 | 4.5290 | 0.4568 |
| K126 | 1.5911 | 4.4803 | 0.4028 |
| E127 |  |  | 0.5363 |
| E128 | 1.6886 | 4.9213 | 0.3233 |
| E133 | 1.8083 | 4.2176 | 0.4382 |
| D134 |  | 4.1494 | 0.5173 |
|  |  |  |  |
| L136 | 1.7687 | 4.2445 | 0.3617 |
| G137 |  |  | 0.3126 |
| F138 | 1.7241 | 2.9994 | 0.1480 |
| S139 | 1.6798 | 2.8161 | 0.3884 |
| L140 | 1.5969 | 3.6860 | 0.0919 |
| F141 | 1.4912 | 1.5069 | 0.3461 |
| G142 | 1.0765 | 1.0784 | -0.3627 |
